# *Weizmannia coagulans* BC99 alleviates hyperuricemia and oxidative stress via DAF-16/SKN-1 activation in *Caenorhabditis elegan*

**DOI:** 10.3389/fmicb.2024.1498540

**Published:** 2024-12-11

**Authors:** Yinyin Gao, Cheng Li, Junfei Li, Mengyao Duan, Xuan Li, Lina Zhao, Ying Wu, Shaobin Gu

**Affiliations:** ^1^College of Food and Bioengineering, Henan University of Science and Technology, Luoyang, China; ^2^Henan Engineering Research Center of Food Microbiology, Luoyang, China; ^3^National Demonstration Center for Experimental Food Processing and Safety Education, Luoyang, China

**Keywords:** *Weizmannella coagulans*, *Caenorhabditis elegans*, hyperuricemia, metabolomics, mechanism

## Abstract

**Introduction:**

Hyperuricemia (HUA) refers to the presence of excess uric acid (UA) in the blood, which increases the risk of chronic kidney disease and gout. Probiotics have the potential to alleviate HUA.

**Methods:**

This study established a hyperuricemia model using *Caenorhabditis elegans* (*C. elegans*), and studied the anti-hyperuricemia activity and potential mechanisms of *Weizmannella coagulans* BC99 (*W. coagulans*) at different concentrations (10^7^ CFU/mL BC99, 10^8^ CFU/mL BC99). Subsequently, we utilized UPLC-Q-TOF/MS to investigate the impact of BC99 on endogenous metabolites in *C. elegans* and identified pathways and biomarkers through differential metabolomics analysis.

**Results:**

The results of this study showed that BC99 treatment significantly reduced the expression of P151.2 and T22F3.3 (*p* < 0.05), reduced the levels of UA and xanthine oxidase (XOD) in nematodes (*p* < 0.05), while extending their lifespan and movement ability (*p* < 0.05). Mechanistically, BC99 activates the transcription factors DAF-16 and SKN-1, thereby inducing the expression of stress response genes, enhancing the activity of antioxidant enzymes and tolerance to heat stress in the body, and reducing the production of ROS (*p* < 0.001). This effect was most significant in the H-BC99 group. Furthermore, non-targeted metabolomics indicated that BC99 predominantly regulated pathways associated with amino acid metabolism (Carnosine), glycerophospholipid metabolism, and purine metabolism.

**Discussion:**

These results underscore BC99 as an effective and economical adjunct therapeutic agent for hyperuricemia, providing a scientific basis for further development and application.

## Graphical Abstract

After intervention, *Weizmannella coagulans* BC99 can activate transcription factors DAF-16 and SKN-1, thereby inducing the expression of stress response genes, enhancing tolerance to oxidative and thermal stress, and reducing the production of UA. Endogenous disorders in hyperuricemic nematodes can be alleviated by regulating pathways related to amino acid metabolism, glycerophospholipid metabolism, and purine metabolism. 
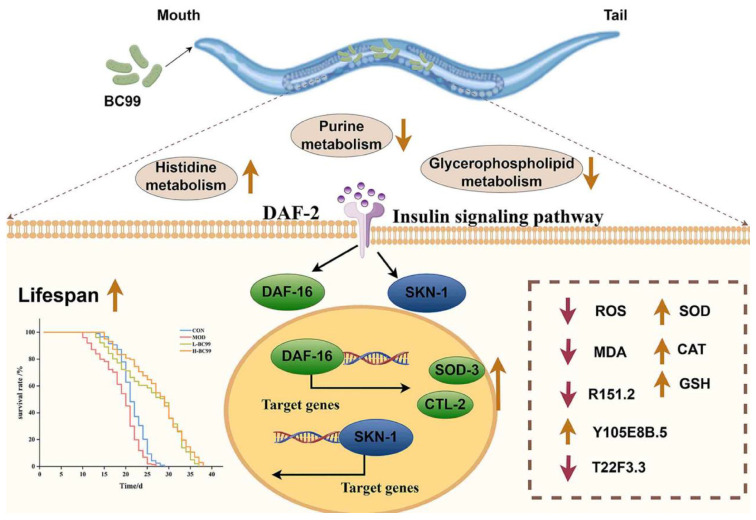

## 1 Introduction

Hyperuricemia (HUA) is a metabolic disorder characterized by elevated uric acid levels due to abnormal purine metabolism (Liu et al., 2022). Long-term HUA could lead to gout and other metabolic diseases such as cardiovascular disease, chronic kidney disease, and diabetes. In recent years, with rapid economic development and lifestyle changes, the incidence of HUA has been increasing annually, becoming a common metabolic disease (Jake et al. 2019). The occurrence of HUA was mainly due to either excessive uric acid production or decreased uric acid excretion in the body. When there was excessive uric acid production in the body, the lack of related enzymes in the purine metabolism process led to uric acid accumulation, thereby posing a risk to health (Ghei et al., 2002). The occurrence of hyperuricemia is closely related to the pro-oxidative state. Oxidative stress is caused by the pathological imbalance between pro-oxidative and anti-oxidative factors. Xanthine oxidase not only catalyzes hypoxanthine to generate xanthine, and then to generate uric acid, but is also considered to be a high producer of superoxide and hydrogen peroxide, thereby generating a large amount of reactive oxygen species (ROS) (Gherghina et al., 2022). Excessive ROS, in turn, can lead to loss of mitochondrial function and accelerate aging (Ma et al., 2021). Currently, the main drugs used clinically to treat HUA include allopurinol (Shijun et al., 2016), febuxostat (Li et al., 2016), and benzbromarone (Azevedo et al., 2019). Although allopurinol is recommended as a first-line drug for treating HUA, it has many side effects, including allergies, gastrointestinal discomfort, and toxic epidermal necrolysis (Lee et al., 2019). Therefore, it is necessary to seek new strategies for treating HUA. In recent years, due to the confirmed beneficial effects of probiotics in regulating human health, attention has also been paid to the relieving effect of probiotics on HUA. Because of its significant efficacy and fewer adverse reactions, probiotics have been widely recognized as a possible choice for treating HUA.

Probiotics are living microorganisms that, when administered in appropriate doses, can have a positive impact on human health (Brian and Nihal, 2017). Increasing evidence suggests that they play a crucial role in gut microbiota and lowering serum uric acid levels. Naruomi et al. (2017) found through animal experiments that *Lactobacillus gasseri* PA-3 degrades inosine and xanthine, thereby reducing serum uric acid levels, thereby lowering uric acid. *Plantarum* Q7 treatment can restore species diversity and improve gut microbiota (Cao et al., 2022a). *Plantarum* LLY-606 significantly reduces serum uric acid levels by inhibiting uric acid secretion and regulating uric acid transport (Shi et al., 2023). Ying et al. (2021) found that *Limosilactobacillus fermentum* can degrade UA in the intestine, thereby reducing the amount of UA accumulated in the intestine, improving intestinal motility, and reducing UA excretion in feces. *Lactobacillus paracasei* X11 has excellent *in vitro* uric acid-lowering activity, can inhibit pro-inflammatory cytokine IL-1β, and regulate adenosine deaminase (ADA), xanthine oxidase (XOD), and transport proteins (GLUT9, NPT1, and URAT1) to normal levels (Cao et al., 2022b). Another study using *Lacticaseibacillus paracasei* MJM60396 to prevent hyperuricemia through various methods such as absorbing purines, inhibiting xanthine oxidase to reduce UA synthesis, and increasing UA excretion by regulating uric acid transport proteins (Lee et al., 2022). These studies suggest supplementing probiotics may be an effective treatment for improving hyperuricemia.

However, traditional probiotics are difficult to survive in extreme environments (Tripathi and Giri, 2014). *W. coagulans* BC99 is a potential new probiotic for the treatment of hyperuricemia, which can improve metabolic disorders directly or indirectly (Osinski et al., 2022). *W. coagulans* has the characteristics of both *Lactobacillus* and *Bacillus*, has good stress resistance, and is resistant to acid and bile salts, and can survive and colonize in the gastrointestinal tract (Drago and De Vecchi, 2009). Clinical studies have found that *W. coagulans* BC99 can improve constipation symptoms in chronic constipation adults and regulate the gut microbiota, and no significant adverse reactions were observed in all patients, which confirms the safety of *W. coagulans* BC99 (Wu et al., 2024). However, the exact mechanism by which *W. coagulans* responds to oxidative stress, reduces uric acid levels, and extends lifespan needs further study. Several reports have shown that *W. coagulans* strains play a potential role in regulating the composition and activity of the microbiota, enhancing immunity, and alleviating metabolic disorders (Zhao et al., 2021). Currently, established animal models of hyperuricemia mainly include mice and rats. However, the uric acid metabolism pathways in rats and mice are significantly different from humans. Uricase is present in most mammals, including rodents, to break down uric acid. However, humans do not have the necessary gene to produce uricase, making them unable to metabolize uric acid. This introduces uncertainties and limitations in modeling. Therefore, relying solely on mouse models to study the mechanisms of anti-hyperuricemia has certain limitations. *C. elegans*, a nematode belonging to the nematode class, is an ideal model for studying lifespan, diseases, and toxicology due to its transparency, small size, short lifespan, and ease of cultivation and manipulation (Shen et al., 2018). As a model organism, *C. elegans* has high value in identifying the activity of hyperuricemia. *C. elegans* lacks uricase and is easy to culture and manipulate, making it an ideal model for studying diseases. The establishment of a hyperuricemia model using *C. elegans* can provide in-depth insights into the signaling pathways and molecular mechanisms that link uric acid to oxidative stress and lifespan. The insulin/insulin-like growth factor signaling pathway (IIS) is a well-known pathway that is involved in stress-activated cytoprotective responses, particularly for the daf-16 (homolog of mammalian FoxO1) transcription factor, as well as activation of downstream genes (Zhang J. et al., 2022). Literature searches have shown that studies have successfully established hyperuricemia models using *C. elegans*, and *C. elegans* lacks uricase in its body (Li et al., 2019). Moreover, there are few studies involving *C. elegans* as a hyperuricemia model, providing a basis for developing more suitable models. Compared to traditional hyperuricemia models, the *C. elegans* hyperuricemia model can provide a more accurate tool for drug screening for hyperuricemia, thus seeking safer and more effective therapeutic drugs.

Therefore, this study established a hyperuricemia model in *C. elegans* with the following goals: (1) to explore whether *W. coagulans* BC99 can reduce uric acid synthesis; (2) to reveal the potential molecular mechanism of *W. coagulans* BC99 in anti-oxidation and reducing uric acid levels and prolonging lifespan; and (3) to combine non-targeted metabolomics to study the biomarkers and potential therapeutic targets of *W. coagulans* BC99.

## 2 Materials and methods

### 2.1 Strains and reagents

*Weizmannia coagulans* BC99 strain was obtained from Wecare Probiotics Co., Ltd. (Suzhou, China). *Escherichia coli* OP50 strain was deposited in the laboratory of the School of Food and Bioengineering. The wild-type strain Bristol N2 and the transgenic strains DAF-2 (CB1370), DAF-16 (GR1307), and SKN-1 (GR2245) were obtained from the Caenorhabditis Genetics Center (CGC, Sao Paulo, MN, USA), and were cultured on *C. elegans* growth medium (NGM) plates with *Escherichia coli* OP50 as the standard food source.

Hypoxanthine and dichlorodihydrofluorescein diacetate (H2DCF-DA) were purchased from Shanghai Aladdin Biochemical Technology Co., Ltd. 5-fluorouracil was obtained from Shanghai yuanye Bio-Technology Co., Ltd. The uric acid assay kit (UA) was purchased from Shanghai Xinfan Biotechnology Co., Ltd. The XOD, SOD, GSH, CAT and MDA kits were purchased from Nanjing Jiancheng Biotechnology Research Institute Co., Ltd. The Total RNA kit, cDNA Synthesis SuperMix for qPCR and Hieff qPCR SYBR Green Master Mix were provided by Hunan Accurate Biological Co., Ltd.

### 2.2 Cultivation, passage and synchronization of *C. elegans*

Following the method outlined by Asthana et al. (2016) with slight modifications, the nematodes were maintained on nematode growth medium (NGM) plates or in liquid S-medium (SM) containing OP50 as a food source. Every 2 days, nematodes were transferred to fresh NGM plates to obtain synchronized populations using the sodium hypochlorite method.

### 2.3 Establishment of high uric acid model in *C. elegans*

The experiment was divided into six groups: the CON group (OP50), the MOD group (OP50 with 0.25 mg/mL hypoxanthine), the L-BC99 group (10^7^ CFU/mL BC99 with 0.25 mg/mL hypoxanthine) (Based on preliminary experiments and literature reports, it was determined that it can effectively induce hyperuricemia without causing excessive toxicity), and the H-BC99 group (10^8^ CFU/mL BC99 with 0.25 mg/mL hypoxanthine). (Since previous studies have shown that both 10^6^ and 10^9^ CFU/mL can affect the normal growth of nematodes, we chose to use 10^7^–10^8^ CFU/mL concentration for intervention) The method referenced was that of Meng et al. (2017) with slight modifications. The synchronized eggs were placed on NGM plates with OP50, L-BC99, and H-BC99 strains. The plates were incubated at 20°C for 46 h to make the nematodes as equal and appropriate as possible. Then they were placed in a six-well plate containing 3.6 mL of SM liquid culture medium. Except for the CON group, the remaining groups were added with 1.2 mL of drug (0.25 mg/mL hypoxanthine) and incubated at 20°C for 24 h to establish a hyperuricemia model.

### 2.4 Determination of XOD and UA activity

Using the method described in section “2.3 Establishment of high uric acid model in *C. elegans*,” after successful modeling, 500 nematodes were collected from each group, the samples were washed three times with M9, thoroughly homogenized with a tissue grinder, centrifuged, and the supernatant was collected. Uric acid levels were determined according to the instructions provided in the Nanjing Jiancheng uric acid and XOD assay kit.

### 2.5 Effect of *W. coagulans* on high uric acid model in *C. elegans*

#### 2.5.1 Lifespan assay

Referring to the method of Kato et al. (2018) with slight modifications, after successful modeling, the *C. elegans* were washed three times with M9 solution. Each experimental group contained 50 individuals of *C. elegans*, which were subsequently transferred to NGM agar plates containing 5-fluorouracil. The death and loss of *C. elegans* were accurately recorded daily using a stereo microscope until all tested *C. elegans* were dead. The standard definition of nematode death was that the nematode no longer responded to external stimuli (such as light touch) and there was no visible muscle contraction activity in the body. In order to exclude false positives, we would conduct multiple observations to confirm the death state of the nematode.

#### 2.5.2 Measurement of athletic ability

Refer to the method of Wang et al. (2018) with slight modifications. Head swing: *C. elegans* were picked onto NGM agar plates without OP50 and observed under a microscope after 2 min. A head swing of 90° by the *C. elegans* was counted as one occurrence. The number of head swings of *C. elegans* was recorded within 1 min. A total of 10 nematodes were picked for observation, and the experiment was repeated in triplicate.

Body bending: Each sinusoidal movement of the *C. elegans*, equivalent to one wavelength, was counted as one occurrence. The number of body bends of the *C. elegans* was recorded within 20 s. A total of 10 nematodes were picked for observation, and the experiment was repeated in triplicate.

### 2.6 *W. coagulans* BC99 effect on ROS activity in *C. elegans* high uric acid model

Refer to the method of Yu et al. (2020) with slight modifications. H2DCF-DA was used as a fluorescence molecular probe to detect the concentration of intracellular ROS in worms. The nematodes from each dose group were washed with M9 buffer and transferred to centrifuge tubes. To each tube, 1 mL of H2DCF-DA solution (10 μmol/L^–1^) was added, and the tubes were placed in a 20°C incubator for a 2 h reaction period (with gentle mixing by inverting the tubes every 30 min). After the reaction, the worms were washed three times with M9 buffer. Subsequently, they were anesthetized with 200 μL of 0.5 mmol/L^–1^ levamisole and mounted on slides. Fluorescence microscopy (Leica Biosystems, China) was used for observation and imaging. Ten nematodes were selected from each group for observation. The fluorescence intensity of ROS was analyzed using Image J1.8.0 software.

### 2.7 Effect of *W. coagulans* BC99 on the antioxidant capacity of *C. elegans* high uric acid model

Using the method described in section “2.3 Establishment of high uric acid model in *C. elegans*,” after successful modeling, collect 500 worms treated with xanthine. The *C. elegans* were collected and washed three times with M9 buffer, followed by thorough homogenization using a tissue grinder. The homogenate was then centrifuged, and the supernatant was collected. SOD (Superoxide Dismutase), MDA (Malondialdehyde), CAT (Catalase), and GSH (Glutathione) levels were determined according to the instructions provided in the Nanjing Jiancheng Uric Acid Assay Kit.

### 2.8 Determination of heat stress ability of mutant *C. elegans*

According to the method in section “2.3 Establishment of high uric acid model in *C. elegans*,” the hyperuricemia model was established using the daf-2, daf-16, and skn-1 mutant. The method of Yin et al. (2024) was used with slight modifications. The mutant nematodes were rinsed thrice with M9 buffer before being placed onto NGM agar plates, with a sub-set subsequently transferred to a constant temperature incubator set at 37°C, at this point, designated as 0 h, the survival status of the nematodes was observed and recorded every 2 h until all nematodes had perished. Each group consisted of 3 plates, with 30 nematodes on each plate.

### 2.9 RNA extraction and quantitative real-time PCR

Each group of approximately 500 worms was collected and washed to extract total RNA. Approximately 1 μg of RNA was extracted and gene expression was detected using a SYBR Green real-time PCR instrument. The studied genes included downstream genes of *daf-2*, *daf -16* and *skn-1*. Primer sequences for all genes are listed in Table 1. The experiment was repeated in triplicate for each condition. Relative expression levels of each gene were analyzed using the 2^–ΔΔCt^ method (Livak and Schmittgen, 2001). The housekeeping gene β*-Actin* was used for normalization of gene expression.

**TABLE 1:** Primers used for PCR and RT-PCR.

Gene	Forward (5′ → 3′)	Reverse (5′ → 3′)
*daf-2*	CGTCAATCGTCAC CGTTTATCTC	GTTATTGGCAATT GACACAGTTCC
*daf-16*	AAAGACAACGAC CAGACGGAAC	ACTGTTCGAATCT CCCTTATCCC
*Skn-1*	ATACTCACCGAG CATCCACCA	TTCTCCATAGCACAT CAATCAAGTC
*Sod-3*	AACTTGGCTAAGG ATGGTGGAG	CCTTGAACCGCAAT AGTGATG
*Ctl-2*	TCCATACCCAGAA GCGTAATCC	TCACATAGATAGCC TTTCCGTCC
*R151.2*	CTGAGCCAGCAAT TCTGAAGTA	TTGAAGCATTGAG ACGAGTGAG
*Y105E8B.5*	ATGCTCGCTC ATCGTGTCCT	GGCGGTCCAGGT CTTCTCAA
*T22F3.3*	GCAGGCTGTGATG GATCGTAAC	TGTGATTGGTGTAG GCGTAGGT
β*-Actin*	GGTTGCCGCTC TTGTTGTAGAC	TACCGACCAT GACTCCTTGATGAC

### 2.10 LC-MS analysis

LC-MS analysis was performed using a UHPLC system (Vanquish, Thermo Fisher Scientific) and the Waters ACQUITY UPLC BEH Amide (2.1 mm × 50 mm, 1.7 μm) liquid chromatography column for the chromatographic separation of target compounds (Wang et al., 2016). Mobile phases consisting of (A) 25 mmol/L ammonium acetate adjusted to pH 9.75 and acetonitrile (B). The injection volume was 2 μL at a sample temperature of 4°C. An Orbitrap Exploris 120 mass spectrometer equipped with an ESI source was used. The sheath gas flow rate and auxiliary gas flow rates were set at 50 Arb and 15 Arb, respectively. The capillary temperature was maintained at 320°C, collision energies were set at 20-30-40 eV (SNCE), and full scan resolution and MS/MS resolution were set to 60000 and 15000, respectively. The experiments were conducted in both positive ion mode (spray voltage 3.8 kV) and negative ion mode (spray voltage −3.4 kV). The raw data was converted to mzXML format using ProteoWizard software, and then metabolite identification was conducted using a collaborative R package. The BiotreeDB (V3.0) database (Zhou et al., 2022) was utilized for this purpose. Finally, visualization analysis was performed using a self-developed R package.

### 2.11 Statistical analysis

The experiments were independently repeated three times. SPSS software was used for analysis, and the results were presented as mean ± standard deviation (SD). Data analysis was performed using Origin 2021 software. One-way analysis of variance (ANOVA) was conducted to assess the significance of differences between means, with significance levels denoted as *p < 0.05, **p < 0.01, ***p < 0.001.

## 3 Results

### 3.1 Effect of *W. coagulans* BC99 on the UA and XOD of high uric acid model in *C. elegans*

**FIGURE 1:**
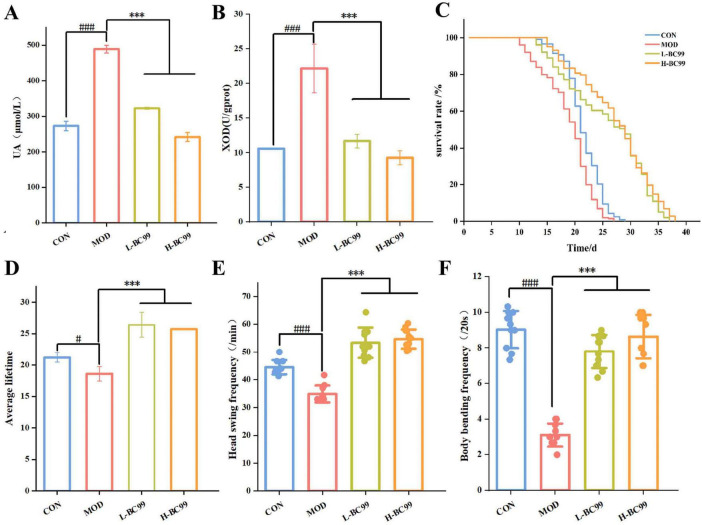
Effect of *Weizmannella coagulans* BC99 on high uric acid model in *C. elegans*. **(A)** UA; **(B)** XOD; **(C)** lifespan; **(D)** average lifespan; **(E)** haed swing; **(F)** body bending. L-BC99 represents the low-dose BC99 treatment group (10^7^ CFU/mL), while H-BC99 represents the high-dose BC99 treatment group (10^8^ CFU/mL). Data are presented as the mean ± SD. Compared with CON group, significant differences in the MOD group are represented by #, ^#^*p* < 0.05, ^##^*p* < 0.01, ^###^*p* < 0.001. Compared with the MOD group, significant differences in the BC99 group are represented by *, *p < 0.05, **p < 0.01, ***p < 0.001.

By establishing a *C. elegans* hyperuricemia model, the efficacy of *W. coagulans* BC99 in reducing uric acid levels *in vivo* was investigated. As shown in Figure 1A, compared to the CON group, the UA level in the MOD group was 488.67 μmol/L, indicating a significant increase in uric acid levels in the nematodes, suggesting the successful establishment of the hyperuricemia *C. elegans* model. Compared to the MOD group, the UA content was reduced to 322.67 ± 3.06 μmol/L after the L-BC99 intervention, and to 242 ± 12.49 μmol/L after the H-BC99 intervention, indicating the potential of *W. coagulans* in reducing uric acid levels. In the process of catalyzing the formation of UA, xanthine oxidase (XOD) plays a crucial role as a substrate. To demonstrate the reduction in uric acid levels with the administration of *W. coagulans* BC99, we determined the content of XOD in each group using a reagent kit. As shown in Figure 1B, compared to the CON group, the intracellular XOD level in the MOD group was 22.16 U/gprot. Compared to the MOD group, the XOD content was reduced to 11.68 ± 0.99 μmol/L after L-BC99 intervention, and to 9.25 ± 1.00 μmol/L after H-BC99 intervention. respectively. It was consistent with the report of Wu et al. (2021). Based on the above results, it was clearly evident that *W.coagulans* BC99 may reduce the production of UA by inhibiting the enzymatic activity of XOD, with H-BC99 showing the most significant effect.

### 3.2 Effect of *W. coagulans* BC99 on the lifespan of high uric acid model in *C. elegans*

The effect of *W. coagulans* BC99 on the lifespan of *C. elegans* in the hyperuricemia model is illustrated in Figures 1C, D. The results demonstrate that the survival curve of worms in the hyperuricemia model group significantly shifted left compared to the CON group (*p* < 0.05). In contrast, the survival curve of worms in the BC99-treated group significantly shifted right compared to the MOD group (*p* < 0.001). Lifespan experiments showed that the average lifespan of nematodes in the MOD group was shorter compared to the CON group, possibly due to the accumulation of excessive uric acid in the nematode’s body, leading to metabolic abnormalities and an increase in uric acid concentration within the nematodes, which had a certain impact on their lifespan. However, the average lifespan of nematodes in the BC99 groups was significantly higher than that in the MOD group. These research findings collectively indicate the positive effects of *W. coagulans* BC99 Bon the *C. elegans* hyperuricemia model.

The ability to move is an indicator of health and lifespan in *C. elegans*. Movement is a coordinated rhythmic activity of the nervous system and muscles. Studies indicate that hydrogen peroxide affects chemosensory neurons and gustatory sensation (Ahluwalia et al., 2016). We evaluated the motor ability of nematodes, as shown in Figures 1E, F, compared to the CON group, the MOD group showed a significant decrease in the number of head swing (34.87 ± 3.05/min) and body bending ability (3.10 ± 0.65/20 s) in the nematodes (*p* < 0.001). After the L-BC99 and H-BC99 interventions, the number of head swing increased to 53.33 ± 5.45/min and 54.67 ± 3.46/min, respectively, and the body bending ability increased to 7.80 ± 0.93/20 s and 8.63 ± 1.22/20 s, respectively, indicating that *W. coagulans* BC99 can enhance the motor ability of nematodes, with this effect being more pronounced at higher doses. Therefore, H-BC99 group was selected for subsequent metabolic profile studies.

### 3.3 Effect of *W. coagulans* BC99 on ROS activity in nematode high uric acid model

**FIGURE 2:**
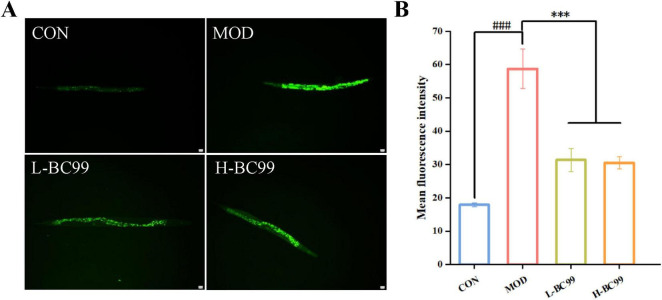
Weizmannella coagulans. *W. coagulans* BC99 enhances the resistance of *C. elegans* to stress conditions (*n* = 10). **(A)** Image of ROS expression in *C. elegans*; **(B)** quantitative fluorescence analysis of worms. L-BC99 represents the low-dose BC99 treatment group (10_7_ CFU/mL), while H-BC99 represents the high-dose BC99 treatment group (10_8_ CFU/mL). Compared with the CON group, ^###^*p* < 0.001. Compared with the MOD group, ***p < 0.001.

According to the free radical theory, the accumulation of excessive free radicals posed significant dangers to various organisms (Ma et al., 2016). To test the *in vivo* antioxidant effect of *W. coagulans* BC99, this study employed the H2DCF-DA fluorescent probe to assess the intracellular reactive oxygen species (ROS) levels in wild-type N2 worms. ROS levels correlate with the fluorescence intensity generated by ROS oxidation, resulting in increased fluorescence of 2′,7′-dichlorofluorescein (Evgeniy and Sergei, 2010). As depicted in Figure 2, compared to the CON group, the relative fluorescence intensity of ROS in the MOD group nematodes increased by 69.47% (*p* < 0.001). Compared to the MOD group, the L-BC99 and H-BC99 groups exhibited reductions of 46.59 and 48.11% (*p* < 0.001), respectively (Figure 2). Therefore, we suggest that *W.coagulans* may act as an antioxidant to ameliorate oxidative damage caused by uric acid by scavenging accumulated free radicals in *C.elegans.*

### 3.4 The impact of *W. coagulans* BC99 on the antioxidant capacity of the hyperuricemia model in *C. elegans*

**FIGURE 3:**
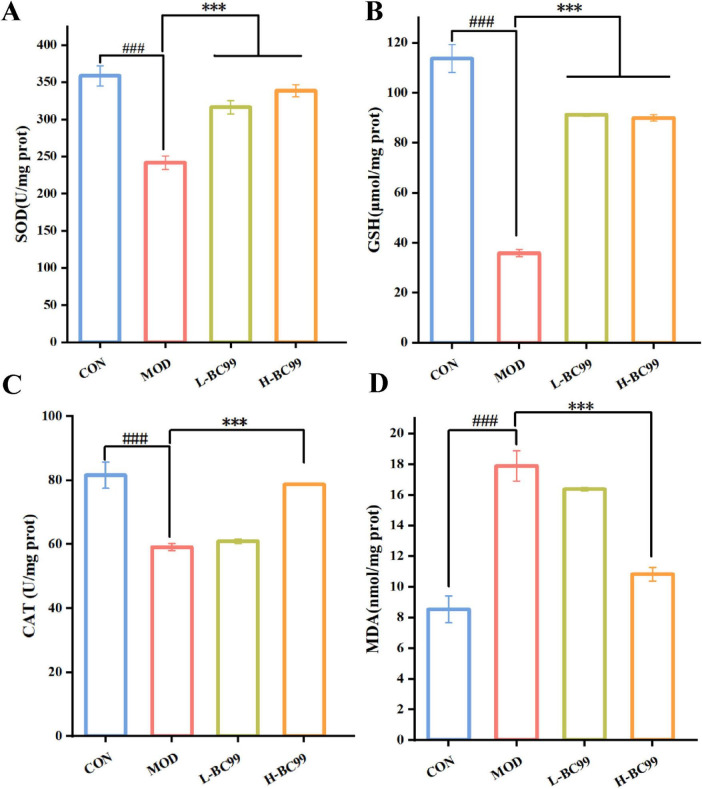
Effects of T *W. coagulans* on the antioxidant capacity of a hyperuricemic nematode model. **(A)** SOD; **(B)** GSH; **(C)** CAT; **(D)** MDA. L-BC99 represents the low-dose BC99 treatment group (10^7^ CFU/mL), while H-BC99 represents the high-dose BC99 treatment group (10^8^ CFU/mL). Compared with the CON group, ^###^*p* < 0.001. Compared with the MOD group, ***p < 0.001.

Maintaining redox homeostasis is essential for the stable functioning of normal physiology. Excessive oxidative stress can lead to unwanted damage when there is an overload of oxidation products, causing a disruption in the body’s adaptation mechanisms (Zhang F. et al., 2022). In In this study, the supplementation of *W. coagulans* BC99 was found to decrease the production of reactive oxygen species (ROS), prompting a further evaluation of the oxidative stress indicators to assess the protective effects of *W. coagulans* BC99 against oxidative stress induced by uric acid. The process of uric acid generation produces reactive oxygen species that can induce oxidative stress. Superoxide dismutase (SOD) eliminates the superoxide anions generated within the organism, while catalase (CAT) constitutes a vital component of the enzymatic system in the body’s antioxidant system, synergistically eliminating free radicals alongside SOD. Glutathione (GSH) protects cellular membrane structure and function from oxidative damage caused by H_2_O_2_. The results from Figure 3 demonstrate that the MOD group led to a significant reduction in SOD, CAT, and GSH levels within the nematodes, accompanied by a notable increase in malondialdehyde (MDA) content (*P* < 0.001). However, this condition significantly improved in the intervention groups with *W. coagulans* BC99, wherein the L-BC99 group, the SOD activity was increased from 241.69 ± 9.03 U/mg prot to 316.33 ± 8.95 U/mg prot, the CAT activity from 59.09 ± 1.09 to 60.91 ± 0.65 U/mg prot, and the GSH activity from 35.79 ± 1.46 to 91.18 ± 0.60 umol/mg prot, while the MDA activity was reduced by 8.43% (*p* < 0.001). In the H-BC99 group, the SOD activity was increased from 241.69 ± 9.03 U/mg prot to 338.52 ± 8.29 U/mg prot, the CAT activity from 59.09 ± 1.09 to 78.76 ± 0.18 U/mg prot, the GSH activity from 35.79 ± 1.46 to 89.92 ± 1.27 umol/mg prot, and the MDA activity was reduced by 39.55% (*p* < 0.001) (Figure 3). The consistency of these results clearly indicates that exogenous *W. coagulans* BC99 counteracts uric acid-induced oxidative stress and confers a defensive effect against uric acid damage in *C. elegans*. This effect was more significant in the H-BC99 group.

### 3.5 The influence of *W. coagulans* on the expression levels of genes in the insulin/IGF-1 signaling (IIS) pathway and uric acid-related genes in *C. elegans*

The *C. elegans* gene *Y105E8B.5*, which is similar to the human gene Hypoxanthine Phosphoribosyltransferase 1 (*HPRT1*), plays a crucial role as an essential enzyme in the purine salvage pathway. Its function includes catalyzing the conversion of xanthine to IMP (inosine monophosphate) and guanine to GMP (guanosine monophosphate) through the transfer of a 5-phosphoribosyl group. Deficiency in this enzyme leads to diminished or absent feedback inhibition, resulting in accelerated purine synthesis and elevated uric acid levels in the body, leading to hyperuricemia.

**FIGURE 4:**
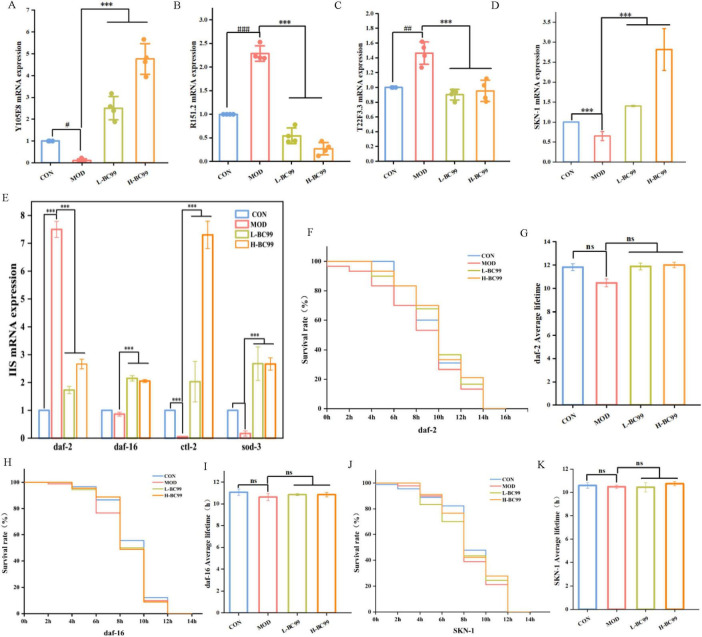
*W. coagulans* the resistance of *C. elegans* to stress conditions and regulates related genes. **(A)** *Y105E.8*; **(B)** *R151.2*; **(C)** *T22F3.3*; **(D)** *skn-1*; **(E)** IIS (*daf-2, daf-16, ctl-2* and *sod-3*). **(F,G)** Survival of DAF-2 (CB1370) under heat stress; **(H,I)** survival of DAF-16 (GR1307) under heat stress; **(J,K)** survival of SKN-1 (GR2245) under heat stress). L-BC99 represents the low-dose BC99 treatment group (10_7_ CFU/mL), while H-BC99 represents the high-dose BC99 treatment group (10_8_ CFU/mL). Compared with CON group, significant differences in the MOD group are represented by #, ^#^*p* < 0.05, ^##^*p* < 0.01, ^###^*p* < 0.001. Compared with the MOD group, significant differences in the BC99 group are represented by *, *p < 0.05, **p < 0.01, ***p < 0.001.

The results of this experiment revealed a significant decrease in *Y105E8B.5* mRNA expression levels in the MOD group (*p* < 0.05). However, following intervention with *W. coagulans*, there was a significant increase in *Y105E8B.5* mRNA levels (*p* < 0.001), indicating a positive impact of *W. coagulans* on hyperuricemia in *C. elegans*. The gene *R151.2*, homologous to the human gene Phosphoribosyl Pyrophosphate Synthetase 1 (*PRPS1*), encodes phosphoribosyl pyrophosphate synthetase, which catalyzes the conversion of ribose-5-phosphate and ATP to phosphoribosyl pyrophosphate (*PRPP*). Increased *PRPP* generation leads to elevated purine synthesis, resulting in increased uric acid production. The gene *T22F3.3* is involved in starch and sucrose metabolism, and its overexpression leads to the production of excess monosaccharides such as fructose from polysaccharides. Fructose has been shown to metabolize into uric acid. The results of this experiment showed a significant increase in the expression levels of the *T22F3.3* mRNA and *R151.2* mRNA in the MOD group (*p* < 0.01). However, following intervention with *W. coagulans*, the expression levels of *T22F3.3* mRNA and *R151.2* mRNA decreased, indicating that *W. coagulans* BC99 can alleviate the production of uric acid (Figures 4A–C).

The above experiments indicate that *W. coagulans* BC99 mediates the expression levels of antioxidant and uric acid-related genes in nematodes to reduce uric acid content. To further confirm the molecular mechanisms of *W. coagulans* on antioxidant pathways in nematodes, RT-qPCR analysis was used to assess the effects of *W. coagulans* BC99 on the transcription factors *daf-2*, *daf-16* and *skn-1*, as well as downstream genes, in the IIS signaling pathway. When *daf-2* is inhibited, its downstream gene *daf-16* shows increased expression in the nucleus, playing an anti-stress role (*p* < 0.05) (Figure 4D). Our results showed that the *daf-16* mRNA in the H-BC99 group increased by 1.02 times compared with the MOD group, the *sod-3* mRNA increased by 1.87 times compared with the MOD group, and the *ctl-2* mRNA increased by 5.74 times compared with the MOD group (*p* < 0.05) (Figure 4D). The transcription factor skn-1 plays an important role in the resistance of *C. elegans* to oxidative stress and prolong lifespan. The *skn-1* mRNA in the H-BC99 group increased by 1.95 times compared with the MOD group (*p* < 0.05) (Figure 4E). The increased expression of these key factors in the insulin signaling pathway indicates that *W. coagulans* BC99 acts by upregulating the expression of antioxidant-related genes and the levels of lifespan-regulating factors, thereby enhancing the nematode’s resistance to oxidative stress and prolonging its lifespan, resulting in a decrease in uric acid levels.

There is a close connection between heat stress and oxidative stress. Heat stress can trigger oxidative stress, affecting the growth, development and health of the body (Mutwedu et al., 2021). In order to verify whether the ability of *W. coagulans* BC99 to resist thermal stress depends on the IIS pathway, we further determined the lifespan of three loss-of-function mutants of *C. elegans*, DAF-2, DAF-1 and SKN-1, under heat stress. The results showed that feeding *W. coagulans* BC99 did not extend the lifespan of the three mutant nematodes (Figures 4F–K), indicating that the heat stress resistance of *W. coagulans* BC99 depends on the participation of DAF-2, DAF-16 and SKN-1 pathways.

### 3.6 Metabonomics analysis

#### 3.6.1 Correlation analysis among QC samples

**FIGURE 5:**
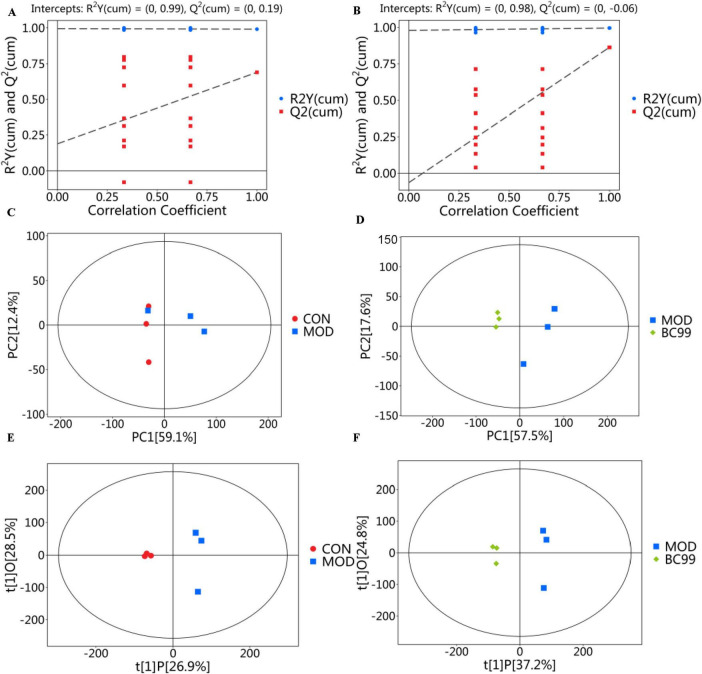
Serum metabolic profiling. **(A)** CON and MOD groups in ESI+ and ESI− mode; **(B)** MOD and BC99 groups in ESI+ and ESI− mode; **(C)** PCA score diagram of the CON group and MOD group in ESI+ mode and ESI− mode; **(D)** PCA score diagram of the MOD group and BC99 group in ESI+ mode and ESI− mode; **(E)** OPLS-DA score diagram of the CON group and MOD group in ESI+ mode and ESI− mode; **(F)** OPLS-DA score diagram of the MOD group and BC99 group in ESI+ mode and ESI− mode.

The correlation heatmap of QC samples illustrates the correlations between QC samples, with each cell color representing the correlation coefficient between one QC sample and another QC sample. A correlation coefficient close to 1 indicates a higher similarity between quality control samples. The results indicate that all QC samples have correlation coefficients > 0.9, suggesting the stability of the measured data in this study and the effectiveness of quality control (Figures 5A, B).

#### 3.6.2 Multi-variate statistical analysis using PCA and OPLS-DA

According to the LC-MS analysis results, principal component analysis (PCA) was employed to study the distribution of metabolites. Figures 5C, D display the PCA score plots for the CON group and the MOD group under the ESI + and ESI- modes. Based on the PCA score plots, *C. elegans* metabolic patterns exhibit variations under different conditions. The study suggests that hyperuricemia disrupts *C. elegans* metabolic pathways. As illustrated in Figure 5D, both PCA score maps revealed a distinct separation of sample points between the CON group and the MOD group, with sample points of the same color indicating a favorable aggregation effect within a specific range. These findings suggest noticeable disparities in serum metabolic components between the CON group and the MOD group, thus indicating the success of the HUA model. The MOD group was completely separated from the BC99 intervention group and closer to the CON group, indicating that BC99 has a certain effect in reducing high uric acid levels. Metabolomics data based on MS (mass spectrometry) are characterized by high dimensionality (many detected metabolite species) and small sample size (few samples detected). These variables include both differential variables associated with categorical variables and a large number of potentially correlated irrelevant variables. This implies that when using PCA for analysis, due to the influence of correlated variables, differential variables will spread across more principal components. This is disadvantageous for discovering genuine inter-group differences and for effectively screening meaningful differential metabolites. Hence, we utilized orthogonal partial least squares discriminant analysis (OPLS-DA) for statistical assessment of the findings. Figures 5E, F depict the principal component analysis OPLS-DA scores for the CON, MOD and BC99 groups. From the figures, it can be observed that the samples from the MOD group are well separated from the CON and BC99 groups, indicating differences in metabolites between the BC99 and MOD groups. This further supports the assertion that BC99 has a significant effect in reducing high uric acid levels.

#### 3.6.3 Analysis of the variance in differential metabolite content and its correlation were also conducted

**FIGURE 6:**
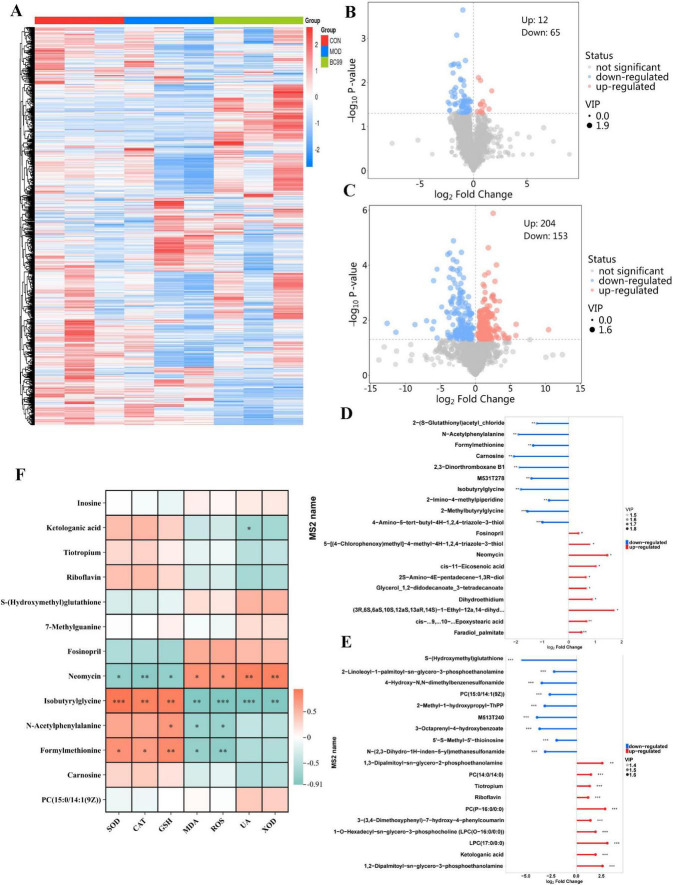
Effect of BC99 on differential metabolites in *C. elegans*. **(A)** Heatmap showing metabolites among different groups; **(B)** volcano plot (CON/MOD); **(C)** volcano plot (MOD/BC99); **(D)** matchstick plot analysis of differential metabolites (CON/MOD); **(E)** matchstick plot analysis of differential metabolites (MOD/BC99); **(F)** correlations between biochemical markers and metabolites. Red indicates positive correlation and blue indicates negative correlation.

As shown in Figure 6A below, this study visualized the changes in annotated metabolites in different groups in the form of a heat map. To visualize the differential metabolites between each control group comparison using volcano plots (Figures 6B, C), 78 (CON VS HUA) and 358 (MOD VS BC99), significant differential metabolites were selected, respectively. Compared to the CON group, intervention with MOD led to the downregulation of 12 differentially metabolized products and the upregulation of 65 differentially metabolized products. Compared to the MOD group, intervention with BC99 led to the downregulation of 204 differentially metabolized products and the upregulation of 153 differentially metabolized products. Subsequently, we calculated the corresponding ratios of the quantitative values of the differential metabolites and took a logarithmic transformation with a base of 2, we took the top 10 up- and down-fold changes to display the results. The horizontal axis shows the logarithmic-transformed change folds, and the color of the points represents the VIP value. This analysis shows the differential metabolites with a large degree of change. In the MOD group, metabolites such as Formylmethionine, Carnosine, N-Acetylphenylalanine, Isobutyrylglycine were down-regulated, and metabolites such as Neomycin, Fosinopril, Glycerol_1,2-didodecanoate_3-tetradecanoate were significantly up-regulated. Carnosine content increased 1.62 times after BC99 treatment (*P* < 0.001). Compared with the MOD group, metabolites such as 7-Methylguanine, 2-Methyl-1-hydroxypropyl-ThPP, S-(Hydroxymethyl)glutathione were significantly down-regulated, and metabolites such as Riboflavin, Tiotropium, Ketologanic acid, PC (P-16.0/0:0) and LPC (17-0/0:0) were significantly up-regulated (*P* < 0.001) (Figures 6D, E). Subsequently, further analysis was conducted to understand the relationship between oxidative stress, biochemical markers, and differential metabolites (Figure 6F). Correlation analysis showed that carnosine, Ketologanie acid, Tiotropium, Riboflavin, Formylmethionine, N-Acetyiphenylalanine and Isobutyrylglyeine were positively correlated with SOD, CAT and GSH levels and negatively correlated with MDA and ROS. Inosine, S-(Hydroxymethyl) glutathione, Fosinopril, Neomyein and PC [15:0/14:1(9Z)] are negatively correlated with SOD, CAT, GSH and positively correlated with MDA, ROS, UA and XOD. Furthermore, 7-Methylguanine and PC [15:0/14:1(9Z)] were found to be positively correlated with UA and XOD.

#### 3.6.4 Potential biomarkers and associated pathways

**FIGURE 7:**
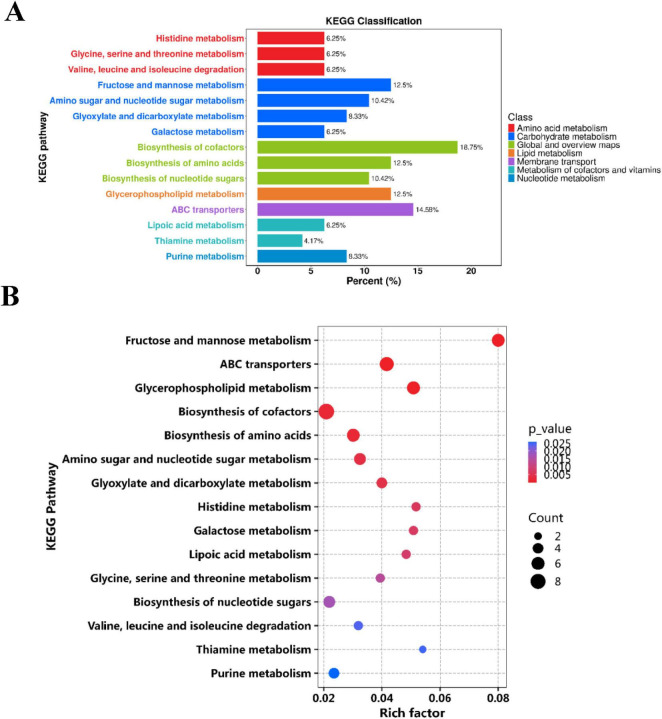
Pathway analysis of significantly altered metabolites. **(A)** KEGG classification diagram of differential metabolites in different groups; **(B)** KEGG enrichment map of differential metabolites in different groups. The abscissa represents the RichFactor corresponding to each pathway, and the ordinate represents the name of the KEGG metabolic pathway. The size of the dots represents the number of differential metabolites enriched in the pathway. The color indicates the size of the *p*-value. The smaller the *p*-value, the redder the color, indicating the more significant the degree of enrichment.

**FIGURE 8:**
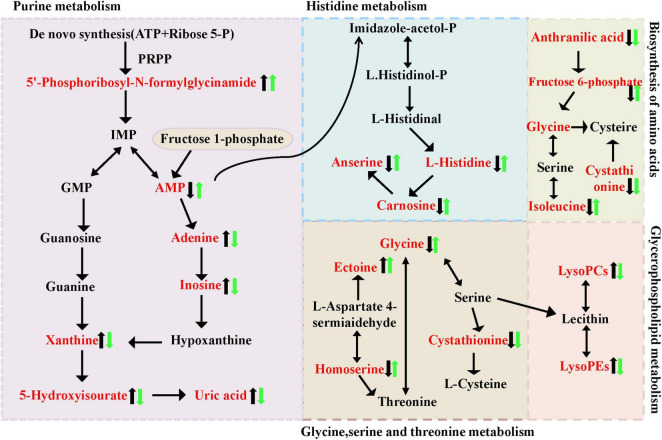
Intervention of *W. coagulans* on the metabolism of MOD mice. The black arrows represent the trend of metabolite changes in MOD mice and the green arrows represent the trend of metabolite shift after BC99 intervention.

Metabolic pathways refer to networks of interactions between metabolites within an organism, reflecting the routes through which compounds are synthesized, broken down, or converted into specific end products via key intermediates. In living organisms, various metabolites collaborate to collectively carry out biological functions. Pathway analysis and annotation can help further explain their functions. A metabolic pathway analysis was performed on the significantly altered metabolites using MetaboAnalyst 5.0. The results revealed differences in metabolic pathways primarily involving Amino acid metabolism (histidine metabolism, glycine, serine, and threonine metabolism), Carbohydrate metabolism (Fructose and mannose metabolism), Global and overview maps, Lipid metabolism (Glycerophospholipid metabolism), Membrane transport, Metabolism of cofactors and vitamins, Nucleotide metabolism (Purine metabolism) (Figures 7A, B). Based on these findings, metabolic pathway Figure 8 was constructed. The results indicate significant disruptions in these pathways in high uric acid nematodes. BC99 was shown to modulate these pathways, thereby regulating endogenous metabolic disorders in *C. elegans*.

## 4 Discussion

Hyperuricemia is a metabolic condition caused by abnormal purine metabolism, resulting in an elevated level of UA (Chen et al., 2021). Regrettably, the negative side effects linked to drugs that reduce uric acid, such as headache, hypersensitivity, and kidney damage, restrict their use in clinical practice (Yu et al., 2016). Therefore, regulating UA metabolism through probiotics has received increasing attention, and there are reports that probiotic supplementation can successfully prevent the accumulation of UA. Cao et al. (2022b) discovered that *Lactobacillus rhamnosus* X11 exhibits strong uric acid-lowering activity *in vitro* and positively contributes to the restoration of gut microbiota. In an animal study, it was found that *Lactobacillus brevis* DM9216, a type of lactic acid bacteria obtained from Chinese sauerkraut, has the ability to break down purine compounds and reduce the level of uric acid in the bloodstream (Wang et al., 2019). Currently, research on hyperuricemia usually establishes rat or mouse models, so establishing a hyperuricemia nematode model is a novel work.

In this study, a nematode hyperuricemia model was established to explore the anti-hyperuricemia activity and mechanism of *W. coagulans*. This study found that after 24 h of xanthine intervention, the levels of UA and XOD in the MOD group increased significantly. This also shows that giving nematodes hypoxanthine (0.25 mg/mL, 24 h) can successfully establish a hyperuricemia model. It was also found that the nematodes in the MOD group. There are phenomena such as shortened lifespan and weakened exercise ability. This is consistent with the study by Wang et al. (2023). Wang et al. (2016) also found that pretreatment of wild-type worms with the LAB strain (*Levilactobacillus brevis* and *Weizmannia coagulans*) increased their survival rate under oxidative and thermal stress conditions by reducing intracellular reactive oxygen levels, significantly extending the lifespan of *C.elegans*. Oxidative stress is involved in the occurrence and development of hyperuricemia. Due to adverse environmental stress, the body produces an excessive amount of reactive oxygen species (ROS), leading to an imbalance between ROS and antioxidant enzymes, causing an oxidative stress response (Nandi et al., 2019). This study found that the ROS content in the MOD group increased significantly, and continuous feeding of *W. coagulans* gradually reduced the excessive production of ROS. The pattern of worm survival, movement, and ROS production suggests that the exogenous Condensed *W. coagulans* the oxidative stress induced by UA and confers a protective effect against UA damage in *C.elegans.* Given that the *W. coagulans* supplement has a positive regulatory effect on ROS production under oxidative stress conditions, it is necessary to track and explain the indicators of oxidative stress to further assess the defensive activity of *W. coagulans* against pathogenic oxidative stress. Oxidative stress in the body often occurs because antioxidants are unable to clear the excessive free radicals, and in this process, SOD, GSH and CAT play important roles. Compared with the CON group, the contents of SOD, GSH and CAT in nematodes in the MOD group decreased significantly, and BC99 intervention restored this trend. FoxO and Nrf2 are two classic transcription factors involved in stress response, reducing oxidative damage and extending the lifespan of mammals. DAF-16 (the C. elegans homolog of mammalian FoxO transcription factor) and SKN-1 (the C. elegans homolog of mammalian Nrf2 transcription factor) participate in the IIS pathway of C. elegans, playing important roles in mediating longevity and resistance to stress (Zhang J. et al., 2022). Under normal circumstances, DAF-16 and SKN-1 are located in the cytoplasm. When cellular homeostasis is disrupted, they translocate to the nucleus, activating genes related to stress response. The beneficial effects of *W. coagulans* BC99 and its interaction with the host in reducing uric acid and promoting health are complex and multifactorial. As a facultative anaerobe, *W. coagulans* BC99 can consume free oxygen in the gut, reducing oxidative reactions and creating an anaerobic and acidic intestinal environment. This helps to inhibit pathogenic bacteria and promote the growth of some beneficial bacteria, maintaining a balanced gut microbiota (Komura et al., 2022). To explore whether the uric acid-lowering effect of *W. coagulans* involves this pathway, we first measured the expression of several key genes in the pathway. We found that *W. coagulans* significantly inhibited the mRNA expression level of the *daf-2* gene and increased the transcription level of *daf-16*. The downstream target genes *sod-3* and *ctl-2* showed increased expression in the nucleus, playing a role in anti-stress, thereby reducing purine accumulation. *skn-1* is a transcription factor in *C. elegans* and is a homolog of the mammalian Nrf2 protein. It plays a crucial role in resisting oxidative stress and increasing the lifespan of *C. elegans*. This study found that intervention with the *W. coagulans* group significantly increased the transcription level of the skn-1 gene. Additionally, through further research on the three gene mutant nematodes of the pathway, DAF-2 (CB1370), DAF-16 (GR1307), and SKN-1 (GR2245), it was found that there was no significant difference in the lifespan of the mutant nematodes under heat stress between the *W. coagulans* group and the MOD group. The above results indicate that *W. coagulans* may enhance the nematode’s resistance to oxidative stress through the insulin pathway genes *daf-2*/*daf-16* and *skn-1*, thereby playing a role in reducing uric acid levels.

PRPS and XOD are closely related to the biosynthesis of uric acid. *PRPS* catalyzes the formation of uric acid from adenosine monophosphate. XOD can convert hypoxanthine to uric acid. An increase in XOD activity leads to the excessive production of uric acid, which the kidneys are unable to excrete, resulting in hyperuricemia. The *T22F3.3* gene is involved in starch and sucrose metabolism, and fructose has been proven to produce uric acid (Lubawy and Formanowicz, 2023). The results of this study found that *W. coagulans* can reduces the mRNA expression levels of the *PRPS* and *T22F3.3* genes, thereby reducing the production of uric acid.

Endogenous metabolism studies indicate that *W. coagulans BC99* primarily affects purine metabolism pathways, amino acid-related metabolic pathways, and glycerophospholipid pathways within nematode bodies. This is consistent with previous studies; Chen et al. (2016) found significant increases in ornithine levels and significant decreases in levels of aspartate, proline, glutamine, serine, pyroglutamic acid, and glutamate in hyperuricemia model rats based on GC-MS combined with serum metabolomics. Li et al.’s (2022) research results indicate that in hyperuricemic mice, there is disruption in purine metabolism, amino acid metabolism, tryptophan metabolism, as well as interactions between neuroactive ligands and receptors. Shan et al. (2021) conducted a non-targeted metabolomic analysis on the plasma of hyperuricemia rats, indicating that the metabolic pathways in hyperuricemia rats involve the biosynthesis of arginine and the metabolism of purines. Uric acid is the final result of purine metabolism in the majority of animals, predominantly produced from hypoxanthine and xanthine through the catalysis of xanthine oxidase (Wang et al., 2021).

Purine metabolism is the process in which adenine and guanine are metabolized in the body through various purine metabolic enzymes into uric acid. Its function is essential for providing energy, regulating metabolism, and producing coenzymes through the purine nucleotide cycle and amino acid metabolism. Purine metabolism includes the *de novo* synthesis pathway, the salvage synthesis pathway, and the catabolic pathway. Among them, the salvage synthesis pathway generates purine nucleotides utilize hypoxanthine, guanosine, and adenine as substrates, and through degradation pathways, adenosine and hypoxanthine are further oxidized to form xanthine and uric acid. As depicted in Figure 8, there are 6 distinct metabolites involved in the purine metabolism process in this study. In comparison to the BC99 group, the MOD group exhibited significantly elevated levels of 5′-Phosphoribosyl-N-formylglycinamide, Adenine, Xanthine, 5-Hydroxyisourate, and UA, while the AMP content was notably decreased, indicating a disruption of the purine metabolism pathway in the MOD group. The MOD group significantly promoted the process of purine metabolism, while *W. coagulans* showed resistance to this effect. It is hypothesized that the increase in AMP in the BC99 group may be linked to histidine metabolism.

Amino acids serve as primary constituents of proteins, and their byproducts are essential for the regulation of protein and energy metabolism. Serving as key regulators in metabolic pathways, amino acids are indispensable for the maintenance of cellular homeostasis. Carnosine, Isobutyrylglycine, N-Acetylphenylalanine, and Formylmethionine are associated with amino acid metabolism. This study found that they are positively correlated with SOD, CAT, and GSH, and negatively correlated with MDA, ROS, UA, and XOD. Some studies indicate that hyperuricemia is associated with histidine metabolism disorders (McCulloch and Marliss, 1975). In histidine metabolism, carnosine, as a major metabolite, significantly decreased in the bodies of high uric acid nematodes. Carnosine possesses various biological activities, including antioxidant, anti-fatigue, anti-aging, and anti-inflammatory effects (McCulloch and Marliss, 1975). For instance, carnosine was discovered to exhibit a shielding impact on hydrogen peroxide-triggered oxidative pressure in the human kidney and substantially alleviated renal damage in diabetic rats (Caruso et al., 2019). Chen et al.’s (2023) research shows that carnosine reduces uric acid levels by inhibiting inflammation and promoting uric acid excretion. However, the intervention with conjugated *W. coagulans* BC99 significantly altered the content of carnosine. The above results show that high uric acid nematodes can cause disorders in the histidine metabolic pathway, and intervention with *W. coagulans* has a certain improvement effect. One of the key pathways by which BC99 may alleviate oxidative stress and UA levels is the production of some metabolites (e.g., carnosine).

There is a certain connection between the metabolism of glycine, serine, and threonine and hyperuricemia. Glycine and serine are precursors of uric acid, and uric acid is a key indicator of hyperuricemia. Therefore, abnormalities in the metabolism of these amino acids may affect uric acid levels.

Phosphatidylcholine is a prevalent and intricate phospholipid present in the human body, widely spread throughout all tissues. In addition to being involved in surfactant and other compound production, it also contributes to bile synthesis, cell signal transduction, and the regulation of body metabolism, thereby ensuring normal body energy and various metabolisms (Wang et al., 2015). In general, the level of LysoPCs in cells or tissues is usually very low. Excessive levels can lead to damage to the cell membrane system and result in serious bodily dysfunction. The findings of this research indicate that the levels of LysoPC [16:1(9Z)], LysoPC [22:5(7Z,10Z,13Z,16Z,19Z)], LysoPC [14:1(9Z)], and LysoPE [16:1(9Z)0:0)] were significantly elevated in the MOD group (*p* < 0.05), suggesting that high uric acid mice cause severe disorders of glycerophospholipid metabolism, and these changes in glycerophospholipids can be used as potential markers. PC and LPC are mainly involved in glycerophospholipid metabolism. This study found that related glycerophospholipid metabolism exists in hyperuricemia nematodes. Consistent with this result, previous studies on the differences in plasma metabolism between hyperuricemia patients and healthy people found that abnormal lipid metabolism is one of the metabolic characteristics associated with hyperuricemia (Yang et al., 2019). Disorders in glycerophospholipid metabolism may cause many diseases. We preliminarily infer that *W. coagulans* may reduce the uric acid content in hyperuricemia by affecting purine metabolism, histidine metabolism, and glycerophospholipid metabolism.

We acknowledge some limitations in translating *C. elegans* findings to human or mammalian models. First, the physiological mechanisms of *C. elegans* are significantly different from those of mammals, which may affect the conservation and comparability of certain signaling pathways. Secondly, the regulatory mechanisms of the DAF-16/SKN-1 pathway may be different in different species, which requires further research to verify. In addition, factors such as environmental factors, nutritional status, and stress response may also affect the function of the DAF-16/SKN-1 pathway, and the expression of these factors may be different in different species. In subsequent experiments, we will replicate our *C. elegans* experiments in mammalian models, including but not limited to *in vivo* and *in vitro* experiments, to verify whether the effects of BC99 on metabolism are consistent across species, explore the metabolic effects of BC99 in more complex organisms, and compare these effects with the observations in *C. elegans* to determine the potential mechanisms of action of BC99 in different organisms.

## 5 Conclusion

In conclusion, this study showed that BC99 can reduce UA and XOD levels in hyperuricemic nematodes. This may be mediated through the *daf-16*/*skn-1* pathway, and BC99 enhances the nuclear localization of the *daf-16* transcription factor, thereby improving stress resistance, reducing intracellular ROS levels and oxidative stress, and regulating lifespan. In addition, BC99 intervention may affect the uric acid content of hyperuricemic organisms by affecting histidine (carnosine) and purine metabolic pathways. Overall, this study reveals the significant potential of *W. coagulans* BC99 in regulating uric acid levels and improving metabolic health. *W. coagulans* BC99 demonstrates a lower risk of side effects and possible long-term health benefits compared with traditional treatments for hyperuricemia. Therefore, *W. coagulans* BC99 may not only serve as an effective treatment option but also has the potential to be used as an adjuvant treatment to improve the overall health and quality of life of patients with hyperuricemia. Future clinical studies will further verify the potential of BC99 in humans.

## Data Availability

The data presented in the study are deposited in the OMIX repository [OMIX - Home], accession number [OMIX008096].
